# Exploring the Potential of Donkey Milk in Enhancing Pneumonia Treatment Outcomes—Pilot Study

**DOI:** 10.3390/antibiotics15020121

**Published:** 2026-01-26

**Authors:** Violeta Kolarov, Anika Trudić, Maja Bogdan, Jovan Javorac, Ivana Čabarkapa, Dragana Tomanić, Ljubiša Šarić

**Affiliations:** 1 Faculty of Medicine, University of Novi Sad, Hajduk Veljkova 3, 21000 Novi Sad, Serbiaanika.trudic@mf.uns.ac.rs (A.T.);; 2 Institute for Pulmonary Diseases of Vojvodina, Put Dr Goldmana 4, 21204 Novi Sad, Serbia; 3 Institute of Food Technology in Novi Sad, University of Novi Sad, Bulevar Cara Lazara 1, 21000 Novi Sad, Serbia

**Keywords:** adjunctive therapy, donkey milk, pneumonia, clinical trial

## Abstract

**Background/Objectives:** Community-acquired pneumonia (CAP) is a major cause of hospitalization and mortality worldwide, with growing interest in adjunctive therapies to enhance treatment outcomes. Donkey milk, long used in traditional medicine for respiratory illnesses, contains bioactive compounds with antimicrobial, anti-inflammatory, and immunomodulatory properties that may benefit patients with CAP. **Methods:** Sixty hospitalized patients were prospectively allocated into two groups based on their consent to receive adjunctive donkey milk supplementation: one received standard antibiotic therapy plus 250 mL of pasteurized donkey milk twice daily for one month, while the control group received antibiotics alone. **Results:** Patients consuming donkey milk showed significantly faster reductions in C-reactive protein CRP and procalcitonin PCT levels, greater radiological improvement (*p* < 0.001), and a shorter average hospital stay (12.46 vs. 14.16 days). Logistic regression analysis identified donkey milk consumption as a significant predictor of shorter hospital stay. Importantly, no adverse effects were reported, and compliance with the supplementation was high. **Conclusions:** These findings suggest that pasteurized donkey milk may serve as a safe and effective natural adjunct to standard antibiotic therapy in managing CAP, with potential to enhance recovery and reduce hospital stay. Further large-scale studies are needed to validate these results and explore broader applications of donkey milk in infectious diseases.

## 1. Introduction

Community-acquired pneumonia (CAP) is defined as an acute infection of the lung parenchyma characterized by at least several symptoms of acute infection, combined with infiltrates on chest X-ray and auscultatory findings in a patient without recent health care exposure [1]. Determining the exact incidence of CAP is challenging, primarily due to differences in case definitions across studies and varying levels of healthcare system development worldwide. Despite that, CAP remains one of the most significant infectious diseases worldwide, contributing substantially to global morbidity and mortality. Recent estimates indicate that its global incidence reached approximately 4350 cases per 100,000 people in 2021 [2]. The disease burden is particularly pronounced among vulnerable populations, including older adults, young children, individuals with compromised immune systems, and patients with chronic underlying conditions [3]. Globally, CAP is the leading infectious cause of death [4], with estimates suggesting approximately 2.2 million deaths globally in 2021 [5], equating to a mortality rate of 0.7 deaths per 1000 individuals per year [6]. The risk of fatal outcomes is higher among hospitalized patients, particularly those treated in intensive care units (ICU), where mortality rates can reach up to 50% [7].

While antibiotics remain the cornerstone of treatment for bacterial CAP, given the associated morbidity, mortality, and economic costs, along with the growing issue of antibiotic resistance, there is increasing research on the effects of various adjunctive therapies aimed at improving treatment outcomes [8]. Beyond modern medical approaches, traditional and natural medicines continue to play an important role in managing respiratory infections in many cultures.

Among these, donkey milk has been used for centuries as a folk remedy for various conditions, including joint pain, gastritis, allergies, gastrointestinal disorders, skin conditions, and respiratory diseases such as pneumonia and bronchitis, particularly in regions like the Balkans, Africa, and parts of the Middle East [9,10]. In recent decades, alongside growing interest in its potential health benefits, donkey milk has gained popularity in Europe, especially in Croatia, France, Hungary, Italy, the Netherlands, and Serbia, as well as in several Asian countries [11].

Often described as a “pharma food,” donkey milk, produced by the female donkey (*Equus asinus*), has gained attention due to its unique nutritional and functional properties. It is rich in proteins, fats, and essential nutrients, offering potential therapeutic benefits, particularly for vulnerable populations such as infants and the elderly [12,13]. Given its nutritional profile, donkey milk holds potential as an adjunctive therapy in managing CAP [14]. Severe CAP often increases the body’s energy requirements [8], which can be supported by the milk’s high-quality protein content. In addition to its general nutritional value, donkey milk contains a variety of biologically active components that have been linked to immune support and antimicrobial activity. These elements, collectively, may contribute to its potential role in supporting recovery during infectious diseases such as CAP [14]. Furthermore, its high content of free fatty acids with antimicrobial activity has demonstrated inhibition of various bacteria, including methicillin-resistant *Staphylococcus aureus* (MRSA), as well as certain fungi [12,13].

Despite its long-standing traditional use for respiratory conditions, scientific evidence supporting the clinical benefits of donkey milk in treating respiratory diseases is lacking. Available data mainly highlight its anti-inflammatory, antiviral, antiproliferative, antitumor, and antimicrobial properties [15,16,17]. While antimicrobial activity against bacterial isolates has been documented in vitro, clear evidence of its clinical efficacy in preventing or treating respiratory infections, including pneumonia, remains insufficient.

Considering the above, the primary aim of this study was to investigate the impact of consuming donkey milk on the clinical outcomes of hospitalized patients with bacterial CAP, including length of hospitalization, rate of reduction in laboratory parameters, rate of radiological regression, frequency of adverse effects, and patient compliance.

## 2. Results

### 2.1. Chemical Composition of Donkey Milk

The basic chemical composition of donkey milk used in this study is presented in Table 1. The milk was characterized by high moisture content, moderate protein, and ash levels.

### 2.2. Participant Characteristics

The study included a total of 60 subjects divided into two equal groups, with an average age of 65.17 ± 10.98 for group I and 63.133 ± 13.06 for group II. The other demographic data are shown in Table 2. Some numerical differences in comorbidity distribution were observed between groups, which may reflect chance variation in a relatively small pilot cohort.

### 2.3. Laboratory Biomarkers: Initial and Follow-Up Values

The results of the T-test show that at admission, all observed values were elevated, with no pronounced differences between the groups. During the follow-up period, a decrease in the values of C-reactive protein (CRP), procalcitonin (PCT), neutrophils, leukocytes and fibrinogen was observed in both groups. However, group I had statistically significantly lower CRP values on the third and seventh days, after discharge and at control compared to group II. PCT values on the third day were statistically significantly different between the groups (0.37 ± 0.70 vs. 1.81 ± 2.68; *p* = 0.005). The values of white blood cells (WBC) (8.51 ± 2.65 vs. 10.00 ± 5.50; *p* = 0.185) and fibrinogen (6.34 ± 1.78 vs. 6.90 ± 2.27; *p* = 0.407) during follow-up period were not statistically significantly different between the groups (Table 3).

Two-factor analysis of variance was used to observe the effects of time, as well as the interaction between groups and follow-up time for the parameters WBC, CRP, PCT and fibrinogen. A significant effect of time was found (F(3,171) = 7.94, *p* < 0.001, partial η^2^ = 0.122), which shows that the number of WBC changed significantly during the follow-up period. However, the interaction between group and time was not significant (F = 2.17, *p* = 0.11, partial η^2^ = 0.037), indicating that the pattern of change over time was not significantly different between groups.

Regarding CRP, a significant effect of time was determined (F(3,174) = 70.12, *p* < 0.001, partial η^2^ = 0.547), which shows a pronounced drop in CRP values during follow-up. The interaction between group and time was borderline significant (F = 3.30, *p* = 0.06, partial η^2^ = 0.054), suggesting a possible difference in the pattern of CRP decline over time between groups.

Observing the effect of time on the change in the values of PCT (F(3,174) = 42.75, *p* < 0.001, partial η^2^ = 0.424) and fibrinogen (F(3,174) = 276.79, *p* < 0.001, partial η^2^ = 0.827), a significant change in the value of the observed parameters was observed during the follow-up period. The interaction between group and time for PCT was significant (F = 4.39, *p* = 0.01, partial η^2^ = 0.070), in contrast to fibrinogen, where it was borderline significant (*p* = 0.057 sphericity assumed) (Table 4).

### 2.4. Radiological Regression Analysis

By analyzing the results of Chi-Squared Tests, it was observed that there is a statistically significant difference in the radiological regression rate between the observed groups (χ^2^ = 16.484; *p* < 0.001) (Table 5).

### 2.5. Logistic Regression Analysis

Binary logistic regression was performed to examine the factors predicting the recovery rate. The results showed a good fit to the data according to the Hosmer–Lemeshow test (χ^2^ = 11.143, df = 8, *p* = 0.194), which indicates that there is no significant deviation between the observed and predicted values. Logistic regression results show that donkey milk consumption was associated with a higher likelihood of shorter hospitalization (≤12 days) (*p* = 0.047; OR = 38.9; 95% CI: 1.05–1448.38); however, the wide confidence interval suggests considerable uncertainty in the magnitude of the effect. The presence of radiological changes significantly increased the chances of prolonged hospitalization (*p* = 0.029; OR = 0.002; 95% CI: 0.00–0.53), while the older age category was at the limit of significance in predicting a longer stay (*p* = 0.051; OR = 0.011). Higher values of PCT and fibrinogen showed a trend towards association with longer hospitalization (*p* = 0.068 and *p* = 0.087, respectively) (Table 6).

## 3. Discussion

To the best of our knowledge, this is the first prospective clinical study to explore the effects of donkey milk consumption as an adjunctive therapy in patients hospitalized with CAP. While traditional use and compositional analyses have suggested antimicrobial and immunomodulatory properties of donkey milk, its clinical application in acute respiratory infections has not been previously documented. Clinical studies are essential to establish the true health benefits of donkey milk in pneumonia management, especially considering that some of its functional properties, including antibacterial activity, may be linked to specific compounds generated during gastric digestion [17]. Consequently, the present findings should be regarded as hypothesis-generating rather than mechanistic, indicating possible supportive roles of donkey milk without establishing a causal relationship.

The observed improvements in inflammatory biomarkers such as CRP and PCT, along with radiological regression in the group receiving donkey milk as supplementary therapy, may reflect the favorable nutritional profile and potential bioactive properties of donkey milk, particularly given that both study groups received comparable guideline-based antibiotic and supportive therapy. However, these findings should be interpreted cautiously, as nonspecific contextual effects and increased oral fluid intake associated with the intervention cannot be fully excluded. The chemical analysis of donkey milk in our study showed 1.86% protein, 0.38% ash, 91.07% moisture, and 8.92% dry matter, which is consistent with previous reports in Serbia indicating protein levels between 1.44 and 1.79%, and 8.82–9.68% dry matter [18]. These values are also similar to findings in Indian gray-breed donkey milk, which reported 1.96% protein, 0.76% fat, 6.30% lactose, and 0.40% ash, suggesting that the chemical composition of donkey milk is relatively stable across breeds, although minor variations may occur due to lactation stage, diet, and environmental factors [19]. These results are further supported by studies from Cyprus, Greece, Italy, and Croatia, which reported protein contents ranging from 1.40% to 1.60% and dry matter between 8.74% and 9.32%, demonstrating that the nutritional composition of donkey milk is largely consistent across different breeds and geographic regions [20,21,22,23]. Such a composition may support its suitability as a natural dietary supplement, providing hydration and essential nutrients, which could contribute to the observed clinical improvements in patients with CAP.

Donkey milk contains several bioactive proteins, including lysozyme, lactoferrin, and α-lactalbumin, which have been reported to exert antimicrobial and immunomodulatory activity primarily in in vitro and experimental settings [24,25,26,27]. For example, lactoferrin exhibits broad-spectrum antimicrobial activity by chelating iron, an essential nutrient for bacterial proliferation, and disrupting biofilm formation, whereas lysozyme and α-lactalbumin compromise bacterial membrane integrity, leading to bacterial lysis [26,28,29]. Interestingly, a study by Šarić et al. [10] reported that raw donkey milk from the Domestic Balkan breed contains significantly higher levels of lysozyme compared to other species, measuring 1.31 g/L [10]. An important consideration in interpreting our results is that the clinical trial was conducted using pasteurized donkey milk. This is particularly relevant given that lysozyme, identified as the dominant antimicrobial and anti-inflammatory component of donkey milk, owing to its significantly higher concentration, is known to be highly thermostable. Literature indicates that lysozyme retains its structural integrity and functional properties at standard pasteurization temperatures (63 °C for 30 min), suggesting that its biological activity remains largely intact in the milk used in this study [30]. However, the current study design does not allow differentiation between direct antimicrobial effects, immune modulation, increased fluid intake, or nonspecific supportive effects, and these mechanisms should therefore be considered plausible explanations rather than confirmed drivers of the observed associations.

Additionally, donkey milk is rich in vitamins A, C, D, and E, which play critical roles in modulating immune responses and reducing oxidative stress, both pivotal in managing bacterial infections like CAP [31]. Vitamins C and E, in particular, act as antioxidants that can mitigate oxidative lung injury caused by excessive inflammation during CAP [32]. Moreover, essential minerals such as zinc and magnesium contribute to enhanced immune function and have been linked to improved clinical outcomes in respiratory infections [33,34]. Free fatty acids present in donkey milk also possess antibacterial and antifungal properties, further supporting its therapeutic potential [35].

Analysis of chest X-ray showed that radiological regression was significantly faster in patients who consumed donkey milk compared to patients who were treated only with standard antibiotic therapy, which aligns with improvements in inflammatory markers. While radiographic resolution in CAP typically lags behind symptom recovery, accelerated improvement may suggest enhanced resolution of alveolar inflammation and exudates, although radiological changes in CAP are influenced by multiple clinical and supportive factors [36]. Similar trends have been observed with other adjuncts like corticosteroids in CAP, where reduced inflammation correlated with quicker radiological clearance [37]. However, unlike pharmacological immunosuppressant, donkey milk offers natural-based approach with low risk of adverse effects, which may be more acceptable for supportive use.

Beyond improvements in biomarkers and radiological regression, patients supplemented with donkey milk experienced a shorter average duration of hospitalization. This reduction may reflect an overall supportive effect comparable to those reported for other nutritional adjuncts, rather than a specific antimicrobial or immunomodulatory mechanism. These results are in line with previous studies showing that nutritional supplements (vitamins C, D, zinc, probiotics) can positively influence pneumonia outcomes [36,38]. For example, vitamin C supplementation has been shown to reduce the length of hospitalization and accelerate clinical improvement in CAP patients [39]. Zinc and vitamin A supplementation has been associated with shorter hospital stays in pediatric CAP cohorts compared to antibiotic treatment alone [40], though comparable studies in adults are lacking. Studies on immunomodulators such as probiotics [41] and certain herbal supplements from Chinese herbal medicine [42] have shown potential benefits in enhancing immune response and reducing symptom duration, though results are variable and further research is needed for definitive conclusions. However, donkey milk’s distinct advantage lies in its unique combination of nutritional, antimicrobial, and immunomodulatory constituents concentrated in a single natural product, offering a multifaceted supportive therapy that may enhance the efficacy of standard care for CAP patients.

Importantly, adverse effects, such as nausea, vomiting, cramps, diarrhea, abdominal pain, and allergic reactions, were not present during donkey milk consumption. This finding aligns with previous research and clinical experience, particularly in pediatric and allergic populations, where donkey milk has been well tolerated. For instance, studies in infants with cow’s milk protein allergy have reported good tolerability rates ranging from 82% to 99%, with minimal reports of systemic allergic reactions [43,44].

Similarly to previous studies of oral micronutrient supplementation and probiotics, donkey milk was well tolerated with no adverse effects reported and high compliance. Prior research consistently highlights zinc and vitamin A as safe in acute CAP—as long as dosing remains moderate—while probiotics also showed good tolerability in hospitalized populations [45]. Moreover, compared to other adjunctive therapies commonly studied in respiratory infections, donkey milk appears to offer a more favorable safety profile. Probiotics, although beneficial in many infectious conditions, have been associated with adverse effects in severely immunocompromised individuals [46]. These findings suggest that donkey milk may represent a well-tolerated and safe adjunctive therapy, particularly advantageous for patients with comorbidities or intolerance to conventional supplementation strategies. Compliance with donkey milk consumption was adequate. This is a critical observation, as introducing new nutritional intervention, especially in hospitalized and vulnerable populations, can often lead to gastrointestinal problems, allergic reaction, or poor patient acceptance [47].

### Limitations and Biological Plausibility

Despite these promising results, several limitations must be acknowledged. First, the sample size was relatively small, and the study was conducted at a single center, which may limit the generalizability of the findings. Second, group allocation was based on patient willingness to consume donkey milk rather than formal randomization, which may have introduced selection bias. Another important limitation is the absence of a placebo-controlled design. Due to the distinctive sensory characteristics of donkey milk, participant blinding was not feasible, and the control group did not receive a placebo intervention. Consequently, nonspecific effects such as expectancy or differential attention cannot be excluded. Furthermore, the lack of blinded outcome assessment—particularly in radiological evaluation—may have introduced observer bias, as chest X-rays were assessed by a single clinician without a standardized scoring system. Moreover, patients in the intervention group consumed an additional 500 mL of fluid daily, which may have influenced certain clinical or laboratory parameters; therefore, the independent effect of donkey milk supplementation cannot be fully disentangled. Numerical differences in comorbidity burden between groups were also observed. Given the pilot nature of the study and the limited sample size, additional analyses adjusting for comorbidities were not performed. Furthermore, the absence of systematic microbiological and radiological subclassification precludes definitive differentiation between typical and atypical pneumonia and limits etiological specificity. Finally, due to the lack of existing clinical trials investigating donkey milk in pneumonia or other respiratory infections, the contextualization of our findings remains limited.

Although the observed clinical effects are consistent with the known properties of donkey milk, direct mechanistic conclusions cannot be drawn from the available data. The study did not include quantitative analysis of individual bioactive components of donkey milk such as lysozyme, lactoferrin, immunoglobulins, vitamins, and free fatty acids before or after freezing and pasteurization. Therefore, while improvements in inflammatory markers, radiological regression, and hospital stay were observed, the underlying biological mechanisms remain speculative. Future studies should include detailed compositional and functional analyses to clarify the mechanistic basis of these effects. Nevertheless, this study provides a foundation for future controlled trials in larger populations.

## 4. Materials and Methods

### 4.1. Sample Collection

Bulk milk samples were obtained from the Special Nature Reserve Zasavica, Serbia. The 15 donkeys of the breed “Domestic Balkan donkey” included in the study, aged between 4 and 10 years, were healthy, well-maintained, and exhibited normal behavior. They were at different stages of lactation, ranging from 75 to 210 days postpartum. Prior to each morning milking session, the udders were rinsed with cold running water and carefully dried with a clean towel. The initial milk streams were discarded to avoid contamination. The collected bulk milk was placed into 0.5 L sterile bottles and immediately frozen after milking. Freezing was performed at −18 °C using standard household-type freezers, which represents routine farm practice in donkey milk production. The milk samples were stored in a refrigerator for 7 days.

### 4.2. Milk Pasteurization

The frozen raw bulk milk was transported in refrigerated containers to the pasteurization facility. The milk was thawed at room temperature immediately prior to pasteurization. A pasteurization regime of 63 °C for 30 min was applied. The milk was pasteurized in 0.5 L glass bottles.

### 4.3. Determination of the Chemical Composition of Donkey Milk

The chemical composition of donkey milk was determined using standard analytical methods. Moisture content was measured by oven drying to constant weight, and dry matter was determined according to AOAC Official Method 925.23 [48]. Protein content was determined using the Kjeldahl method according to AOAC Official Method 991.20 [49], while ash content was analyzed following the procedure described by Carić et al. [50]. All analyses were performed in triplicate, and results are expressed as percentages (%). All measurements were conducted at the Institute of Food Technology in Novi Sad, University of Novi Sad.

### 4.4. Ethical Approval and Informed Consent

Participation in the study was voluntary. Before enrollment, all participants were verbally informed about the study’s aim and provided with “Participant Information,” detailing the research design. Afterward, patients signed the “Informed Consent” form for participation in the study. The research was approved by the Ethics Committee of Institute for Pulmonary Diseases of Vojvodina (IPDV), decision number 2-II/2021-1, on 22 September 2021.

### 4.5. Study Design

This prospective clinical study investigated the clinical outcomes of using donkey milk as a supplementary therapy in treating CAP with presumed bacterial etiology in patients hospitalized at the IPDV, Serbia, from June 2022 to January 2024. Sixty patients diagnosed with CAP based on clinical presentation, physical examination, chest radiographs and laboratory blood tests were included. All patients who met the inclusion criteria were consecutively screened for eligibility.

After receiving detailed verbal and written information about the study, including the proposed adjunctive consumption of pasteurized donkey milk, patients were invited to participate. Group allocation was based on patient consent to consume donkey milk as an adjunct to standard therapy. Patients who agreed to donkey milk supplementation (250 mL of pasteurized donkey milk twice daily for one month, sourced from the Special Nature Reserve “Zasavica”, Serbia) were assigned to the intervention group (Group 1, *n* = 30), while those who declined formed the control group (Group 2, *n* = 30) and received standard therapy alone.

All patients in both study groups received standard-of-care treatment for CAP in accordance with contemporary international clinical guidelines [1]. Empirical antibiotic therapy was initiated upon hospital admission and administered parenterally in all patients. The antibiotic regimens included a combination of a third-generation cephalosporin (ceftriaxone) plus a macrolide (azithromycin), a respiratory fluoroquinolone as monotherapy, or a combination of ceftriaxone and a respiratory fluoroquinolone. The choice, duration, and escalation of antibiotic therapy were guided by clinical response and standard institutional practice and were not influenced by group assignment. Non-antibiotic standard supportive measures for CAP, including oxygen therapy when indicated, bronchodilators, antipyretics, intravenous fluids, and thromboprophylaxis, were applied according to institutional protocols and did not differ systematically between groups.

CAP was diagnosed based on a compatible clinical presentation (acute onset of cough, fever, dyspnea, and/or pleuritic chest pain, and pathological auscultatory findings), the presence of new pulmonary infiltrates on chest radiography, and supportive laboratory findings. Prior to commencing antibiotic therapy and donkey milk consumption, all participants underwent standard laboratory testing such as blood tests for differential blood count and inflammation markers (WBS, CRP, fibrinogen and procalcitonin). Sputum samples for microbiological culture were obtained in patients with productive cough when feasible; however, no cultures yielded a definitive bacterial pathogen. This likely reflects limitations of routine sputum sampling in CAP, including prior antibiotic exposure and sample quality. Radiological findings included focal or multifocal alveolar infiltrates consistent with typical pneumonia, without systematic radiological subclassification (e.g., lobar versus bronchopneumonia).

Laboratory analyses were repeated every three days to evaluate the differences in inflammation marker reduction rates between groups. Weekly chest X-rays monitored radiological improvement rates. Chest radiographs were evaluated at baseline, during hospitalization, and at follow-up as part of routine clinical care. Radiological regression was assessed based on visual comparison with baseline imaging. Complete radiological regression was defined as the resolution of pulmonary infiltrates initially consistent with pneumonia, with no residual consolidation visible on follow-up chest radiography. Incomplete regression was defined as partial persistence of infiltrates. Radiological assessments were performed by a single experienced clinician involved in patient care. No formal standardized radiological scoring system was applied, and assessments were not blinded to group allocation. For both study groups, follow-up continued for one month after the start of hospitalization, with regular check-up on the twentieth day after discharge. During each visit, laboratory examinations, and chest X-rays were performed.

Adverse effects, such as nausea, vomiting, cramps, diarrhea, abdominal pain, and allergic reactions, were monitored during donkey milk consumption. These effects were recorded by the study team during hospitalization and by patients post-discharge in a diary, reported during regular check-ups visit. Compliance with donkey milk consumption was similarly monitored.

### 4.6. Eligibility Criteria

Inclusion criteria for the participation in the study were patients > 18 years, hospitalized at IPDV, and diagnosed with presumed bacterial pneumonia, based on clinical, radiological, microbiological and radiological data. Exclusion criteria included refusal to participate, liver cirrhosis, acute pancreatic and gallbladder disease, acute bleeding ulcer, terminal renal insufficiency, acute surgical conditions (including pleural empyema), impaired swallowing, recent malignancy (last 5 years), celiac disease, lactose intolerance, milk allergy, pregnancy, and lactation.

### 4.7. Statistical Analysis

Descriptive statistics were used for data processing in order to analyze the characteristics of the patients. Then, to determine the existence of a difference in laboratory parameters between the two analyzed groups, a T-test was applied. In order to examine the effect of donkey milk consumption on the change of inflammatory parameters during hospitalization and the follow-up period, a two-way ANOVA with repeated measurements was conducted. Bonferroni-adjusted *p*-values were used for post hoc analyses following the repeated-measures ANOVA. To examine factors predicting the recovery rate, binary logistic regression was performed including variables related to group (donkey milk consumption and control group), presence of chronic diseases, gender, age and inflammatory parameters at discharge. For the purpose of logistic regression, hospitalization duration was dichotomized using a cut-off value of ≤12 days, which corresponded to the median length of hospital stay in the study population and was considered clinically relevant for distinguishing shorter versus prolonged hospitalization.

## 5. Conclusions

Taking into account all available literature data as well as the results of our research, we can confirm the hypothesis of a potentially positive impact of donkey milk consumption on clinical, laboratory and radiological parameters in patients treated for CAP, as well as a significant impact on the speed of recovery and length of hospitalization. While these findings are encouraging, further clinical investigations are needed to confirm these results and explore the broader therapeutic applications of donkey milk in infectious diseases. Given the potential risk of zoonotic transmission, pasteurized donkey milk should be considered the recommended and safe form for human consumption. Although pasteurized donkey milk was used in this study, future research may explore alternative processing approaches, such as lyophilization or non-thermal technologies, which could help preserve heat-sensitive bioactive compounds while maintaining microbiological safety. While key components such as lysozyme remain stable under heat treatment, optimized processing methods may further enhance the milk’s therapeutic potential without compromising consumer safety.

## Figures and Tables

**Table 1:** Chemical composition (%) of donkey milk.

Parameter	Content (%)
Protein	1.86
Moisture	91.07
Ash	0.38
Dry matter	8.92

**Table 2:** Descriptive statistics for all participants.

Parameters		Group I	Group II	*p*-Value
Age		65.17 ± 10.98	63.133 ± 13.06	0.603
Duration of hospitalization		12.46 ± 3.5	14.16 ± 3.7	-
Sex	men	19 (63%)	19 (63%)	1.000
	women	11 (37%)	11 (37%)	
Comorbidities	0	2 (6.67%)	7 (23.33%)	0.320
	1	8 (26.67%)	4 (13.33%)	
	2	11 (36.66%)	9 (30%)	
	3	9 (30%)	10 (33.34%)	

Note: *p*-values refer to between-group comparisons at baseline. Duration of hospitalization represents an outcome variable and was therefore not included in baseline statistical testing.

**Table 3:** Results of T-test (Mean ± SD) for measured parameters by group and time point.

Parameter	Time Point	Group 1 (Mean ± SD)	Group 2 (Mean ± SD)	*p*
CRP	Admission	207.44 ± 132.62	228.86 ± 121.53	0.568
	3rd day	75.6 ± 63.4	119.6 ± 89.8	0.05
	7th day	18.3 ± 23.7	41.9 ± 40.7	0.034
	Discharge	6.2 ± 7.0	14.0 ± 13.0	0.009
	Control	3.7 ± 5.0	6.7 ± 5.9	0.033
WBC	Admission	13.10 ± 4.11	12.92 ± 5.27	0.933
	3rd day	8.51 ± 2.65	10.00 ± 5.50	0.185
	7th day	7.67 ± 2.45	8.74 ± 3.52	0.215
	Discharge	7.59 ± 2.73	7.76 ± 2.99	0.672
	Control	7.69 ± 2.02	7.39 ± 2.04	0.4
PCT	Admission	2.65 ± 4.69	4.69 ± 6.79	0.175
	3rd day	0.37 ± 0.70	1.81 ± 2.68	0.005
	7th day	0.07 ± 0.12	0.41 ± 1.03	0.069
	Discharge	0.04 ± 0.06	0.04 ± 0.05	0.854
	Control	0.02 ± 0.00	0.02 ± 0.00	0.561
Fibrinogen	Admission	7.75 ± 2.02	8.41 ± 2.52	0.344
	3rd day	6.34 ± 1.78	6.90 ± 2.27	0.407
	7th day	4.46 ± 1.23	5.09 ± 1.59	0.164
	Discharge	3.74 ± 0.93	4.08 ± 1.07	0.294
	Control	280.76 ± 156.8	340.00 ± 127.2	0.702

**Table 4:** Results of Two-Way Repeated Measures ANOVA for WBC, CRP, PCT, and fibrinogen (time × group).

Parameters		F	df	*p*-Value	Partial η^2^	Sphericity Correction
WBC	Time	7.94	3, 171	<0.001	0.122	None
	Time × group	2.17	2.21, 125.10	0.112	0.037	Greenhouse–Geisser
CRP	Time	70.12	3, 174	<0.001	0.547	None
	Time × group	3.30	1.28, 74.14	0.061	0.054	Greenhouse–Geisser
PCT	Time	42.75	3, 174	<0.001	0.424	None
	Time × group	4.39	2.40, 138.90	0.010	0.070	Greenhouse–Geisser
Fibrinogen	Time	276.79	3, 174	<0.001	0.827	None
	Time × group	2.56	1.58, 58.00	0.111	0.042	Greenhouse–Geisser

Note: When the assumption of sphericity was violated, Greenhouse–Geisser correction was applied. Exact F-values, degrees of freedom, and *p*-values are reported.

**Table 5:** Analysis of radiological regression in both groups of patients.

Chest X-Ray		Upon Discharge	Follow Up
milk (N)	complete regression	18	23
	incomplete regression	12	7
control (N)	complete regression	3	9
	incomplete regression	27	21
Chi-Squared Tests (χ^2^)		16.484 (*p* < 0.001)	13.125 (*p* < 0.001)

**Table 6:** Logistic regression of individual predictors within group I.

Predictor	B	SE	Wald	*p*	OR [Exp(B)]	OR 95% CI
Group I	3.662	1.845	3.941	0.047	38.936	1.05–1448.38
1 comorbidity	−3.727	3.020	1.523	0.217	0.024	0.00–8.95
2 comorbidities	−4.797	2.889	2.758	0.097	0.008	0.00–2.38
3 comorbidities	−2.163	2.735	0.625	0.429	0.115	0.00–24.47
4 comorbidities	1.204	2.462	0.239	0.625	3.334	0.03–415.52
Sex	−1.975	1.584	1.555	0.212	0.139	0.01–3.09
Age ≤ 40	−3.641	2.564	2.016	0.156	0.026	0.00–3.99
41–65	−4.523	2.320	3.800	0.051	0.011	0.00–1.02
WBC	−0.177	0.202	0.768	0.381	0.838	0.56–1.24
CRP	−0.164	0.121	1.857	0.173	0.848	0.67–1.08
PCT	−5.019	2.753	3.324	0.068	0.007	0.00–1.46
Fibrinogen	−1.455	0.850	2.932	0.087	0.233	0.04–1.23
X ray	−6.285	2.886	4.744	0.029	0.002	0.00–0.53
Constant	34.653	15.380	5.076	0.024	–	–

Note: ORs are presented with 95% confidence intervals. Wide confidence intervals reflect the pilot nature of the study and the limited sample size.

## Data Availability

The data used to support the findings of this study are available in the present manuscript.
